# Best Practice in Surgical Treatment of Malignant Head and Neck Tumors

**DOI:** 10.3389/fonc.2020.00140

**Published:** 2020-02-12

**Authors:** Christian Simon, Piero Nicolai, Alberto Paderno, Andreas Dietz

**Affiliations:** ^1^Service d'Oto-rhino-laryngologie – Chirurgie cervico-faciale, Centre Hospitalier Universitaire Vaudois (CHUV), Université de Lausanne (UNIL), Lausanne, Switzerland; ^2^Department of Otolaryngology—Head and Neck Surgery, University of Brescia, Brescia, Italy; ^3^Department of Otolaryngology, Head and Neck Surgery, University Leipzig, Leipzig, Germany

**Keywords:** head and neck cancer, paranasal sinus, skull base, quality assurance, surgery, best practice

## Abstract

**Purpose of review:** Defining the best practice of surgical care for patients affected by malignant head and neck tumors is of great importance. In this review we aim to describe the evolution of “best practice” guidelines in the context of quality-of-care measures and discuss current evidence on “best practice” for the surgical treatment of cancers of the sino-nasal tract, skull base, aero-digestive tract, and the neck.

**Recent findings:** Current evidence based on certain structure and outcome indicators, but mostly based on process indicators already helps defining the framework of “Best practice” for head and neck cancer surgery. However, many aspects of surgical treatment still require in-depth research.

**Summary:** While a framework of “Best practice” strategies already exists for the conduction of the surgical treatment of head and neck cancers, many questions still require additional research in particular in case of rare histologies in the head and neck region.

## Introduction

Defining “best-practice” of surgical care for head and neck cancer patients is of utmost importance (1). The purpose of this article is to first summarize the evolution of such “best-practice” guidelines in the context of quality assurance (QA) programs for head and neck cancer surgery. Secondly, we will outline current evidence to be considered for “best-practice” in the field of sino-nasal/ skull base, upper-aerodigestive tract, and neck surgery. Data and views provided in this review will help to define, what should be considered “best-practice” in the field of head and neck surgery in the future.

## Evolution of Quality Assurance for Head and Neck Cancer Surgery

### History

The very first surgical quality improvement program was created in 1994 by the Veterans administration (VA) health system in North America (2). It consisted of the simple reporting of morbidity and mortality. For a longer period no further action was taken, until in 2001 the Institute of medicine (IoM) of the United States published an article with the title “Crossing the quality chasm,” in which it was demanded to take action to further improve the quality of surgical care in the United States (US) (3). As a result, the American College of surgeons (ACS) and the Veterans Administration health system created the national surgical quality improvement program (NSQIP). An additional political dimension was gained, when the ACS submitted in 2005 a three-phase improvement program to the US House of Representatives (4). This program was revised in 2007 focusing mainly on process indicators as the main indicators to act on (5). ACS-NSQIP is today the largest QA program for surgery in North America.

Surgical QA programs outside the US developed later. In 2014 the European cancer audit (EURECCA) was created by several European societies including the European Organization for Research and Treatment of Cancer (EORTC), the European Organization for Surgical Oncology (ESSO), the European Society for Radiotherapy and Oncology (ESTRO), and the European Society for Medical Oncology (ESMO) (6). Two years later EORTC together with ESSO and the Japanese Clinical Oncology Group (JCOG) founded a surgical care program called SURCARE. This program however had a more academic goal in aiming for high-quality standards in surgical clinical research (7). Another international society worth mentioning is the Society for enhanced recovery after surgery (ERAS). This society develops guidelines for perioperative patient care. Such guidelines have been published for head and neck free tissue transfer (8). ERAS protocols have been evaluated previously and demonstrated to improve quality of care, patient-reported and operative outcomes, and patient safety. They also help reduce costs (9).

### Components and Confounders of a Quality Assurance Program

In 1966, Donabedian defined the components of a QA program. It consists of indicators allowing for measuring certain aspects of structure, processes, and outcomes (10). Structure herein refers to the characteristics of the healthcare system, the facilities, and hospital infrastructure. Processes are surgical procedures and perioperative treatment. Outcome refers to the results of the healthcare experience. This can be various survival endpoints (11). While structure and process indicators are typically dependent on the institution and/or physician, other variables influencing outcome of the patient are rather patient-driven, i.e., age, comorbidities, performance status, stage of disease, severity of intervention needed etc. (12). These variables need to be taken into consideration, since they serve as confounders and impact on the results of a quality program. Efforts have been made to identify such confounders with an impact on i.e., post-operative complications and various risk-calculators and even neural networks for risk-stratification have been developed (12, 13).

### Critical Structure, Process, and Outcome Indicators

The number of patients a hospital is treating for a particular disease is commonly referred to as “patient volume.” This is an important structure indicator. With respect to head and neck cancer patients high-volume hospitals have been demonstrated to provide a lower long-term mortality. The same holds true for the number of patients seen per physician commonly referred to as physician volume. Also the volume per physician has impact on long-term mortality if it comes to head and neck cancer patients (14).

Certain of these indicators have impact on survival only, if examined in the context of a particular tumor site. In a study on oral cavity cancer the “appropriate referral to radiation therapy” was found to be significantly associated with overall survival (OS), disease specific survival (DSS), and disease free survival (DFS) (15). However, for laryngeal cancers “pre-treatment multidisciplinary evaluation” was important for survival (16). A recent analysis based on the national cancer data base (NCDB) revealed that the delay to adjuvant therapy was associated with higher mortality (17). Adherence to guidelines from the national comprehensive cancer network (NCCN) to initiate adjuvant postoperative radiation therapy within 6 weeks was found to vary widely between institutions (18, 19). Therefore, continued performance monitoring is important to follow the implementation of clinical pathways (20). This monitoring can be assured by providing feedback to health care providers on performance indicators. This was recently demonstrated in a “post-feedback” cohort of head and neck cancer patients, where an improvement of the surgeon's performance next to a reduction of the length-of-stay of patients was observed (21).

Process and outcome indicators for surgical oral cavity cancer patients were recently reported. Besides a nodal yield upon neck dissection (≥18), return to the operating room within 2 weeks, and re-admission within one month were associated with OS, DSS, and DFS (15). Also for laryngeal cancers nodal yield was impacted on survival (16). Another process indicator of importance with impact on mortality is obtaining a negative surgical margin (17).

## What is “Best Practice” in Head and Neck Surgery: Sino-nasal and Skull Base Surgery

Under the umbrella term of “Cancer of the sinonasal tract and skull base” (CSTSB) a galaxy of rare histologies characterized by a wide variability of biological behavior is included. In recent years, this peculiarity led to an emphasis on the role of histology, apart from the site of origin and size of the lesion, in the decision-making process to select the ideal sequence of treatments (“histology-driven approach”) (22). Surgery, which remains a fundamental step in the treatment pathway, currently offers a wide spectrum of procedures, ranging from minimally invasive, purely endoscopic approaches to extensive open resections needing complex reconstruction. In this view, it is essential to precisely define “best practice” in the management of CSTSB to offer an optimal treatment approach to each patient and render outcomes homogeneous across different centers.

However, in consideration of the unique profile of CSTSB (i.e., rarity and histologic heterogeneity), the absolute scarcity of clinical trials, and the lack of specific high level of evidence data, it is extremely difficult to formulate “best practice” guidelines. One example is the American College of Radiology Appropriateness Criteria for cancers arising in the nasal cavity and paranasal sinuses, which rate the suitability of diagnostic and treatment procedures (23). A second example is represented by two documents on chordoma: a position paper on management guidelines (24) and a recent update on best practices for management of local-regional recurrent lesions (25). To get an idea of the paucity of data on CSTSB from well-conducted studies, a review of 71 clinical trials on skull base tumors published in 2017 showed that 83.1% investigated treatments for pituitary tumors, 15.5% for vestibular schwannomas, and 1.4% for sino-nasal/anterior skull base tumors. Furthermore, only 7.7% of trials included surgery (26).

Taking into consideration the main components of a quality assurance platform, as defined by Donabedian (10) (structure, process, and outcome), it is possible to identify several critical factors that play a role in determining treatment results in each of these settings.

“*Structure*” refers to the characteristics and facilities of the healthcare institution. Patient volume represents the most important factor influencing survival in this category. In fact, the expertise of the surgeon and multidisciplinary team are critical when dealing with rare and diverse tumors. However, data attesting improved survival in patients treated in high-volume centers are available only for head and neck cancer in general, with no specific information on CSTSB (14, 27). Of note, in this case, patient volume refers to the experience not only of treating physicians (i.e., surgeon, radiation oncologist, and/or medical oncologist), but also of other specialists involved in the diagnostic process and post-treatment surveillance. In fact, a dedicated and experienced head and neck radiologist is essential to adequately guide therapeutic decisions and follow-up strategies. Similarly, the experience of a dedicated head and neck surgical pathologist directly has an impact on adequate definition of the disease, and consequently, on the most appropriate treatment strategy. This has been demonstrated by several studies on tumors of the sino-nasal tract, showing that re-evaluation in high-volume institutions of biopsies revealed diagnostic errors in 10–23.8% of cases. (28–30). In this view, the International Collaboration on Cancer Reporting has devised specific guidelines aimed at improving and standardizing pathology reporting in sino-nasal cancer (31).

Finally, surgical approaches to the skull base and paranasal sinuses, especially endoscopic ones, require dedicated instruments and facilities. A multidisciplinary team should be able to prevent or manage each unexpected sequela or complication with specific tools (e.g., trans-nasal Doppler probe, hemostatic agents) and collaboration with different departments (i.e., neurosurgery, interventional radiology, intensive care unit).

Considering the “*process*” of patient management, “best practice” dictates some recommendations and quality measures that should be applied and evaluated in both the pre- and post-operative phases.

As a general rule, biopsy should be performed after adequate imaging (computed tomography, magnetic resonance, or both) to avoid complications related to unexpected hypervascular lesions or meningoencephaloceles. The procedure may be performed under local or general anesthesia; however, it is essential to obtain an adequate tissue volume, since unrepresentative biopsies may lead to misdiagnosis even when evaluated by experienced head and neck surgical pathologists. A recent paper suggests that this concept holds especially true when endoscopic and imaging findings suggest a high-grade malignancy (32).

Tumor excision with negative margins is the principal aim of oncologic surgery, and has been identified as one of the main metrics of the quality of surgery (17, 33). In CSTSB, achievement of this goal may require that the surgical team switches from an endoscopic to an external procedure, but involvement of vital structures (i.e., internal carotid artery, cavernous sinus) may sometimes lead to incomplete resection (R1-R2). However, when compared to all the other head and neck mucosal sites, the definition of “clear margins” for CSTSB is controversial and their assessment is hampered by a series of factors. In trans-nasal endoscopic surgery, resection of tumors is often performed through step-by-step disassembly of the lesion starting from the endonasal portion and moving to the periphery, so that assessment of margins is typically made on the most external layer of resection (i.e., dura, periorbita) and samples taken from the surgical bed (i.e., nasal, naopharyngeal, and/or septal mucosa). In external procedures as maxillectomies, an “en-bloc” resection is typically achieved. However, in view of the complexity of the anatomy together with the frequent presence of necrosis and mobile bony fragments, the correct orientation of the specimen with labeling of anatomic structures is of utmost importance to obtain proper evaluation of margins. However, this evaluation is typically dichotomic (yes or no), and no specific data on the millimetric definition of “free” or “close” margins do exist. A different scenario is encountered in tumors like chordoma and chondrosarcoma, where assessment of resection is not based on margin status, but according to intraoperative and post-operative radiologic evaluation. The absence of any visible tumor corresponds to “Gross Total Removal (GTR).” In spite of all these limitations and differences, several recent publications reporting the results of trans-nasal endoscopic surgery for sino-nasal cancer or clival chordoma reiterate the positive impact on prognosis of achieving negative margins or GTV, respectively (34, 35).

Furthermore, a process indicator that is relevant to all surgical procedures, including dural resection, is post-operative cerebrospinal fluid (CSF) leak. It is well-known that this complication can be influenced by several factors: location and size of the defect, communication with a cistern or ventricle, previous radiotherapy, and type of tissues used for reconstruction. Nonetheless, this variable should be regarded as an important quality metric and carefully monitored.

Finally, in view of the histopathologic variety and multidisciplinary management of CSTSB, non-surgical treatments should be precisely intertwined with surgery, with adequate indications and timing. In this regard, the delay between the surgical procedure and adjuvant (chemo)-radiotherapy also represents a strong indicator of the quality of treatment and has been identified as a significant prognosticator in head and neck cancer.

With respect to survival “*outcomes*,” most series available in the literature are burdened by significant biases: they have frequently focused on a single treatment approach but include multiple histologies. However, treatment choice is predominantly histology-driven.

## What is “Best Practice” in Head and Neck Surgery: Upper Aero-digestive Tract and Neck

More than 80% of resectable head and neck tumors are squamous cell carcinomas situated in the oral cavity, oro- and hypopharynx and larynx (HNSCC). Best practice in surgery of HNSCC depends on the profound knowledge of surgical principles and a sufficient surgical experience. It consists of performing resections with clear pathological margins > 5 mm (R0) and obtaining good functional/esthetic outcome and quality of life, which is based on the appropriate choice of reconstruction (36).

Furthermore, best practice in head and neck surgery is associated with a multidisciplinary approach reflecting tumor board decisions and thinking in multimodal concepts combining surgery, oncology, and radiation oncology if needed. John “Drew” Ridge underscored this imperative in his presidential lecture “We show pictures, they show curves” at the AHNS annual meeting in 2010. He stated the need of an interdisciplinary education of head neck surgeons:” This is the only way that the future ‘multidisciplinary team’ will have not merely head and neck surgeons, but rather head and neck surgical oncologists as members; that is what I hope the guidelines come to reflect in years to come” (37). Recently Liu et al. (38) demonstrated that multidisciplinary tumor boards have a positive impact on head and neck cancer patient outcome, but further literature addressing questions of best practice in this field is lacking.

Moreover, within the “Choosing Wisely Canada” campaign, first recommendations of best practice in diagnostics in head and neck cancer have been published (39). Additionally, sentinel node biopsy in patients with oral cancer has been discussed comprehensively in the literature and surgical consensus guidelines have been published recently (40).

Retrospective data based on p16 testing show that HPV16 positive oropharyngeal cancer patients have a better survival prognosis than HPV16-negative regardless of their treatment, i.e., primary surgery or chemo-radiation (41). It is therefore not yet any adequate to discontinue any surgical treatment approaches to this disease, before clinical prospective trials have not clearly determined detrimental effects of surgery in this disease. Moreover, treatment de-escalation trials including non-surgical and surgical treatments are on the way, assessing the role of minimally invasive surgical techniques (transoral laser microsurgery: TLM, trans oral robotic surgery: TORS) to minimize functional deficits in HPV16 positive disease.

In 2009 the outcomes report from a multi-institutional retrospective trial was utilized by the United States Food and Drug Administration (FDA) to approve the use of the da Vinci Surgical System. TORS procedures have been described to manage pathologies at numerous anatomic sites from the glottis and hypopharynx to the nasopharynx and skull base (42). Today, there are no data showing superiority of surgical over non-surgical treatment in HPV-positive oropharyngeal carcinoma. TORS has gained clinical relevance also outside the oropharynx (43) owing to the competition between companies involved in the development of new transoral tools (44).

An older but well-established transoral technique to remove even larger but still accessible tumors of the upper aerodigestive tract is the transoral laser microsurgical (TLM) method, in which the tumor can be taken out in pieces, with precise visualization and control of the margin (45–50). This technique is well-established as part of routine treatments in many centers worldwide and useful in nearly all head and neck locations.

Furthermore, older techniques like open partial and total laryngectomies, laryngo-pharyngectomies, lateral pharyngectomies, and the broad spectrum of open surgery for the mandible, maxilla, and oral cavity have still a relevant place in the treatment of head and neck cancer and should belong to a curriculum, which should be part of a state-of-the-art head and neck surgical education. It is therefore not yet any adequate to discontinue any surgical treatment approaches to this disease, before clinical prospective trials have not clearly determined detrimental effects of surgery in this disease.

Modern techniques of reconstruction are strongly linked to the success of a surgical procedure. Potential defects and postoperative functional and cosmetic results should be discussed by both the patient and the surgeon. In addition, an oncological sound resection must be performed, meaning the surgeon must not compromise the completeness of the excision of the tumor, even if a larger or more challenging defect for a reconstruction may result. Besides pedicled flaps, microvascular free tissue transfer offers distinct advantages in head and neck reconstruction in particular for scalp, facial, oral cavity, osteo-cutaneous, and pharyngeal defects (51–54).

A notable technical advancement in microsurgery has been the introduction of perforator flaps (55). The great advantage of perforator flaps is a decreased donor site morbidity, better adaptation to the reconstructive challenge, and improved aesthetic outcome (56).

The treatment of the neck has been classified by Robbins (57) describing the different types of neck dissections. Neck dissection is a routine part of any head and neck surgical concept and can be neglected only in T1 N0 glottic cancer. This has been underscored by the results of a randomized controlled prospective trial comparing elective and therapeutic neck dissections in node-negative early-stage oral cancer demonstrating significantly higher rates of overall and disease-free survival in the elective neck dissection group (58).

Quality assurance in free flap reconstruction is strongly linked to failure rate and failure emergency surgery and should be benchmarked by comparing outcomes (59). Other surgical and medical complications, like unplanned tracheostomies, revision surgery for any reason, primary and secondary emergency hospital admission and factors linked to risk of in-hospital death should also be benchmarked based on national data sets for instance. An “Informatics-based Framework for Outcomes Surveillance (IFOS)” in Head and Neck Surgery has been proposed recently (60, 61).

Compared to sino-nasal and skull base surgery, literature on best practice in head and neck surgery of other locations is limited to guidelines, recommendations, and evidence related to controlled trials comparing mostly conservative therapy concepts, but not surgical techniques specifically. The problem of forced clinical implementation of new surgical techniques (i.e., TORS) without sufficient evidence from RCTs has been addressed already (62).

## Conclusion

In the years ahead, the scientific community contributing to the evolution of management of sino-nasal and skull base cancer has the challenge and responsibility to collect a sufficient volume of high-quality data to answer open questions. This will help in the definition of “best practice” guidelines for surgery of CSTSB.

“Best practice” in head and neck surgery requires the concentration of such procedures in centers providing strict quality assurance based on certification processes. Moreover, center criteria like participation in clinical trials and transparency of clinical outcome should be mandatory for high quality patient care.

## Author Contributions

CS, AD, and PN: conceptualization. CS, PN, AD, and AP: formal analysis, investigation, and writing. CS: supervision.

### Conflict of Interest

The authors declare that the research was conducted in the absence of any commercial or financial relationships that could be construed as a potential conflict of interest.
